# Lymphedema complicated by protein-losing enteropathy with a 22q13.3 deletion and the potential role of *CELSR1*: A case report

**DOI:** 10.1097/MD.0000000000026307

**Published:** 2021-06-18

**Authors:** Song Xia, Zhong Liu, Huifang Yan, Kun Chang, Yuguang Sun, Jingmin Wang, Wenbin Shen

**Affiliations:** a Department of Lymphatic Surgery, Beijing Shijitan Hospital, Capital Medical University; b Department of Pediatrics, Peking University First Hospital, Beijing, China.

**Keywords:** 22q13.3 deletion syndrome, *CELSR1*, intestinal lymphangiectasia, primary lymphedema, protein-losing enteropathy

## Abstract

**Introduction:**

22q13.3 deletion syndrome is a well-known syndrome characterized by typical clinical findings including neonatal hypotonia, absent or severely delayed speech, intellectual disability, and other various features, and detection of a heterozygous deletion of chromosome 22q13.3 with the involvement of at least part of *SHANK3*. It is reported that 10% to 29% of patients with 22q13.3 deletion syndrome present lymphedema. Protein-losing enteropathy (PLE) has never been reported in 22q13.3 deletion syndrome.

**Patient concerns:**

The patient presented to our institution for refractory hypoalbuminemia and chronic lymphedema in both legs.

**Diagnosis:**

The patient manifested intellectual disability, absent speech, tooth grinding, dysmorphic face, and abnormal hands and toenails. Copy-number variation sequencing confirmed the maternal deletion in 22q13.31-q13.33 (chr22:46285592–51244566, hg19). The patient was genetically diagnosed with 22q13.3 deletion syndrome.

**Interventions:**

Low-fat diets and medium-chain triglycerides supplements were prescribed. The patient was recommended to wear compression garments and elevate legs.

**Outcomes:**

The symptom of diarrhea was resolved, but hypoalbuminemia persisted. Lower extremities lymphedema was gradually becoming severe.

**Conclusions:**

Primary lymphedema and PLE can occur simultaneously in a patient with 22q13.3 deletion syndrome. The 2 phenotypes could share the same genetic etiology of congenital lymphatic abnormalities. *CELSR1* deletion may play a role in lymphatic dysplasia. The case also provides additional proof of the pathogenic effect of *CELSR1* on hereditary lymphedema.

## 1 Introduction

22q13.3 deletion syndrome, also named as Phelan-McDermid syndrome, is a well-known syndrome characterized by typical clinical findings including neonatal hypotonia, absent or severely delayed speech, intellectual disability, and other various features, and detection of a heterozygous deletion of chromosome 22q13.3 with the involvement of at least part of *SHANK3*.^[1]^ It is reported that 10% to 29% of patients with 22q13.3 deletion syndrome present with lymphedema,^[1–4]^ which is a recognized feature in many syndromes and can be caused by primary lymphatic dysplasia resulting from genetic defects.^[5]^ However, the underlying molecular basis of lymphedema in 22q13.3 deletion syndrome remains to be explored.

Protein-losing enteropathy (PLE) is characterized by hypoproteinemia due to the loss of protein in the gastrointestinal lumen. A variety of etiologies can lead to PLE, including intestinal lymphangiectasia and congenital malformations of lymphatics.^[6]^ To date, PLE has not been reported in patients with 22q13.3 deletion syndrome.^[1,2]^

Here, we describe primary lymphedema accompanied with PLE in 1 Chinese girl with 22q13.3 deletion, in which *CELSR1*, a potential novel disease-causing gene of hereditary lymphedema was noted.^[7]^ We speculate that *CELSR1* may be the genetic causative factor for primary lymphedema and PLE presented in 22q13.3 deletion syndrome.

## 2 Case presentation

### 2.1 Ethics statement

Written informed consent to participate was obtained from the parents of the patient. Genetic tests were approved by the Medical Ethics Committee of Peking University First Hospital (No. [2005]004).

### 2.2 Clinical features and investigations

The 20-year-old female patient presented to our institution for refractory hypoalbuminemia and chronic lymphedema in both legs. Lymphedema in her left lower extremity and right hand accompanied with developmental delay, absent speech, and diarrhea was noted at the age of 2 years. The biochemical test showed hypoalbuminemia. Low-fat diets and medium-chain triglycerides supplements helped alleviate the symptoms of diarrhea and lymphedema. Compression garment wear and limb elevation were suggested. After that, the symptom of diarrhea was resolved, but hypoalbuminemia persisted. Lower extremity lymphedema was gradually becoming severe. The patient needed regular albumin transfusion to alleviate hypoalbuminemia.

**Figure 1:**
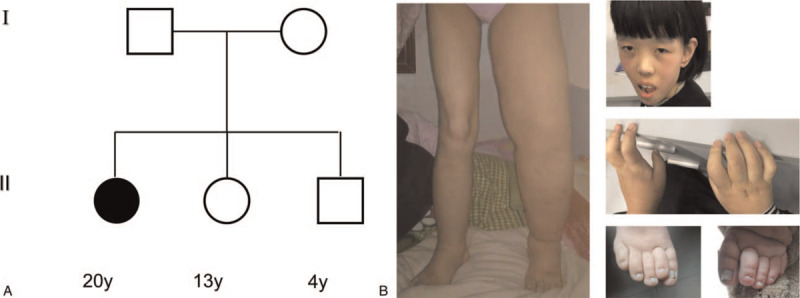
The pedigree and clinical features of the proband. (A) The proband has normal parents and siblings. (B) The proband presents lymphedema in the thick left lower limb, dysmorphic face, large and fleshy hands, and dysplastic toenails.

Upon physical examination, intellectual deficiency, the absence of speech, tooth grinding, dysmorphic face with prominent ears and wide nasal bridge, large and fleshy hands, dysplastic toenails, thick left leg with pitting edema (Fig. 1A and B), and left lower lung dullness were noted.

**Figure 2:**
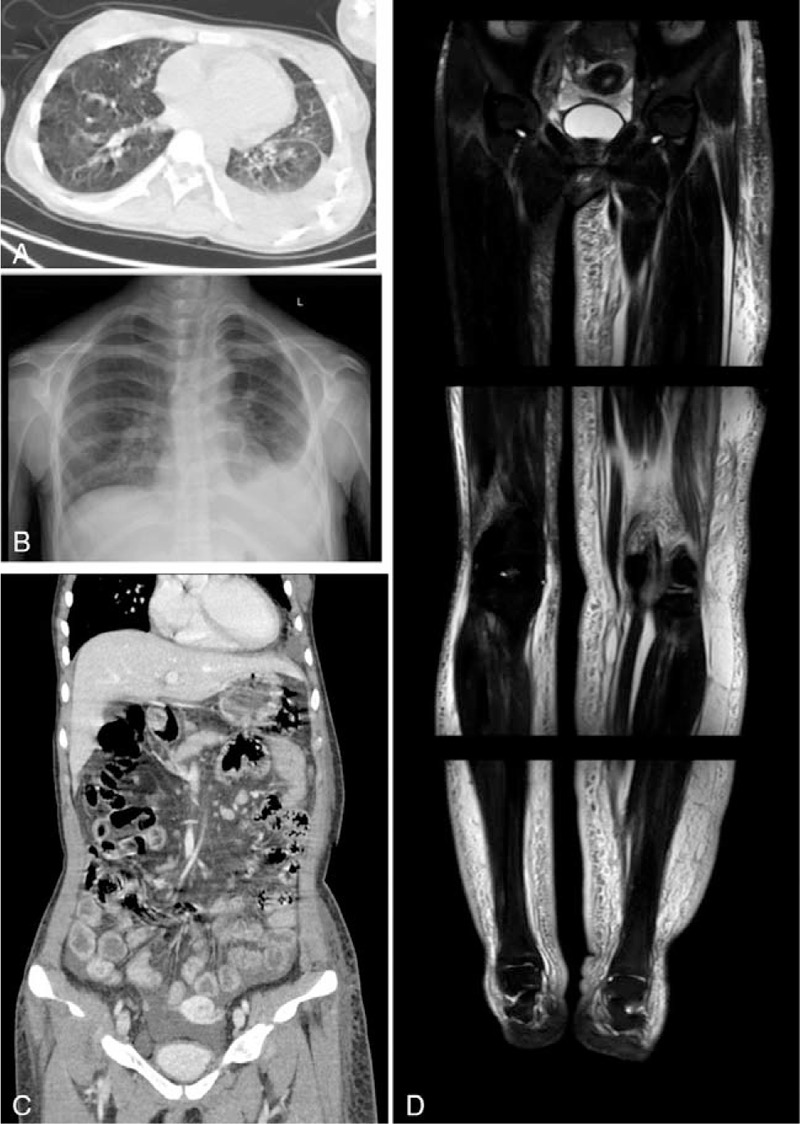
Radiographs of the proband at 20 years old. (A) and (B) Pleural effusions. (C) Thickening small intestinal wall, mesenteric edema, and minor ascites. (D) Magnetic resonance in legs show a subcutaneous deposit of lymph and fibrosis of adipose tissue.

Laboratory studies revealed lower lymphocyte percentage (16.5%), lower albumin (24.8 g/L) and immunoglobulin level (IgG 6.24 g/L, IgA 0.76 g/L, and IgM 0.49 g/L), normal auto-immunological test, elevated CA-125 (321.7 U/mL) and CYFRA21–1 (8.99 ng/mL) level, elevated thyroid-stimulating hormone level (7.53 uIU/mL), normal thyroid hormone level, normal liver enzyme level, negative urine protein, and normal estimated glomerular filtration rate (137 mL/min/1.73 m^2^). The cardiac test showed a normal echocardiograph, long QT/QTc interval (430/451 ms), and incomplete right bundle branch block. Left pleural effusion, thickening of the small intestinal wall, mesenteric edema, and ascites were seen in the computed tomography scan (Fig. 2A to C). Magnetic resonance showed lymphedema in the lower extremities. There were subcutaneous deposits of lymph and fibrosis of adipose tissue in the left leg (Fig. 2D). Non-contrast magnetic resonance lymphography could not be performed because the patient could not follow instructions. Lymphangiography under general anesthesia was refused by the parents considering the risk of pulmonary infection. All related investigations did not suggest any renal, cardiac, or hepatic causes of edema and hypoalbuminemia.

### 2.3 Lymphoscintigraphy

**Figure 3:**
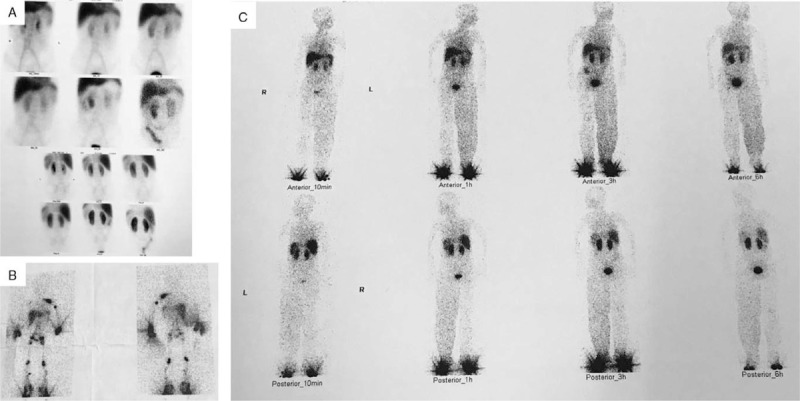
^99m^Tc-labeled dextran lymphoscintigraphy and ^99m^Tc-labeled human serum albumin scintigraphy. (A) Intestinal loss protein imaging documented protein loss into the intestinal lumen in the right lower quadrant of the abdomen. (B) Lymphoscintigraphy shows the absence of lymphatic drainage vessels in the right upper limb and lymph nodes in the right axillae. (C) Lymphoscintigraphy shows lymphedema in both legs, thickened left leg, the absence of lymphatic drainage in lower extremities, and lymph nodes in the popliteal fossa and above the bilateral inguinal ligaments.

^99m^Tc-labeled dextran lymphoscintigraphy and ^99m^Tc-labeled human serum albumin (HSA) scintigraphy were performed in the proband. ^99m^Tc-labeled HSA scintigraphy documented protein loss in the intestinal lumen (Fig. 3A). Although fecal α-1 antitrypsin clearance determination was not performed, the diagnosis of PLE was rational. The photo from lymphoscintigraphy when the patient was a 2-year-old showed the absence of lymphatic drainage in the right upper limb and lymph nodes in the right axillae (Fig. 3B). The other bipedal lymphoscintigraphy in the 20-year-old showed lymphedema, thickened left leg, the absence of lymphatic drainage routes in lower extremities, and lymph nodes in the popliteal fossa and above the bilateral inguinal ligaments (Fig. 3C), which suggested primary lymphatic dysplasia.

### 2.4 Whole-exome sequencing and copy-number variation sequencing

**Figure 4:**
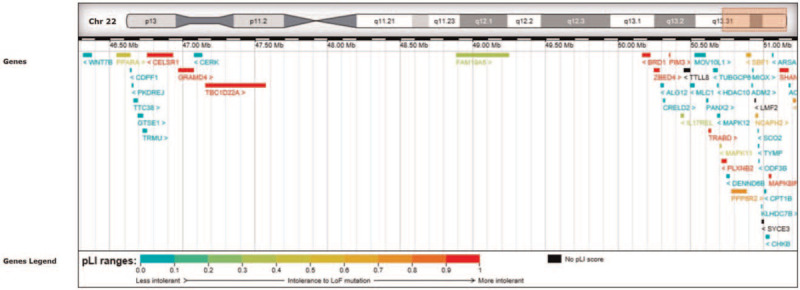
CNV-seq. CNV-seq confirmed the maternal deletion in 22q13.31-q13.33 (chr22:46285592–51244566, hg19). *CELSR1* (chr22:46756731–46933067, hg19) is included by the deletion according to the data from DECIPHER. CNV-seq = copy-number variation sequencing.

Trio-based whole-exome sequencing (WES) and low coverage parallel copy-number variation sequencing (CNV-seq) were performed. No promising SNV or indel variants in known disease-causing genes recorded in OMIM stood out in WES analysis. Interestingly, some rare variants that did not conform to the law of Mendelian inheritance were noticed. Three rare variants *TRABD*:c.39C>A, *HDAC10*:c.880G>A, and *CELSR1*:c.7061G>A are homozygous, heterozygous, and wild type in the proband, father, and mother, respectively. All these 3 genes are located on 22q13.31-q13.33, which suggests the occurrence of maternal uniparental disomy or deletion of this segment. WES-based CNV analysis and further CNV-seq confirmed the maternal deletion in 22q13.31-q13.33 (chr22:46285592–51244566, hg19) (see Fig. 4).

The deletion region encompasses 49 genes, 10 of which (*SCO2*, *TYMP*, *SHANK3*, *SBF1*, *ARSA*, *TUBGCP6*, *MLC1*, *TRMU*, *ALG12*, and *CHKB*) are known to be associated with human disease. The disorders caused by *SCO2* and *SHANK3* are autosomal dominant genetic diseases, while those disorders associated with the remaining 8 genes present with the autosomal recessive pattern. No rare deleterious variants in these 8 recessive genes were noted by re-analysis of WES data. Of 10 genes, *SHANK3* was the only one sensitive to gene dosage. According to the ACMG standards,^[8]^ the deletion was classified as pathogenic with a score of 2.8 (≥0.99, pathogenic).

A gene list associated with PLE and primary lymphedema was built based on references from Online Mendelian Inheritance in Man, PubMed, and Web of knowledge (Table 1). By comparing the 49 genes with our established gene list related to PLE and primary lymphedema, *CELSR1* was noticed (see Fig. 4).

**Table 1:** Established gene list related to protein-losing enteropathy and primary lymphedema. Genes associated with protein-losing enteropathy and primary lymphedema were retrieved from OMIM, PubMed, and Web of knowledge.

Genes related to primary lymphedema
*GJC2, VEGFC, FLT4, EPHB4, PIEZO1, FOXC2, ADAMTS3, FAT4, CCBE1, Angpt2, Efnb2, ITGA9, Elk3, Nrp2, Pik3r1, Prox1, Lcp2, NRAS, RIT1, PPP1CB, SOS1, RAF1, MRAS, BRAF, SHOC2, RRAS2, KRAS, PTPN11, SOS2, LZTR1, GATA2, SOX18, TSC1, TSC2, AKT1, KIF11, CBL, GJA1, PTPN14, IKBKG, PIK3CA, CELSR1, HGF, HRAS, RASA1, MET*

Genes related to protein-losing enteropathy
*PLVAP, DGAT1, CCBE1, FAT4, ADAMTS3, PIEZO1*

## 3 Discussion

As a recurrent microdeletion syndrome, 22q13.3 deletion syndrome has been well characterized and *SHANK3* has been designated as the key gene to elucidate the neurological symptoms like developmental delay and autistic-like behavior. Except for the neurological system, other systems including skin, endocrine, immune, urogenital, and lymphatic system are also involved in some patients,^[1,4]^ which may attribute to different genomic content in the deletions. Our case manifested intellectual disability, absent speech, tooth grinding, dysmorphic face, and abnormal hands and toenails, which is consistent with the phenotype of 22q13.3 deletion syndrome. Genetic analysis revealed a *de novo* heterozygous deletion in 22q13.3 encompassing 49 genes, inclusive of *SHANK3*. Given the consistent, well-defined phenotype and the pathogenic variation, the patient was genetically diagnosed with 22q13.3 deletion syndrome.

Of note, progressive lymphedema complicated by PLE was the main complaint of the patient. Lymphoedema is reported in 10% to 29% of patients with 22q13.3 deletion syndrome^[1–4]^ and it can be accompanied by chylous pleural and peritoneal fluids.^[9]^ PLE has never been reported in 22q13.3 deletion syndrome.

It is known that PLE could be secondary to lymphatic abnormalities.^[10]^ Generalized lymphatic dysplasia (MIM# 616843) and Hennekam lymphangiectasia-lymphedema syndrome (MIM # 235510, MIM # 616006, MIM # 618154) could present with lymphedema and intestinal lymphangiectasia. It is suspected that primary lymphedema and PLE in our patient shares the same genetic etiology of congenital dysplasia of lymphatics.^[6]^ Gonzalez-Garay et al^[11]^ reported that the proband with an early inactivating mutation in *CELSR1* displayed lymphatic backflow and tortuous lymphatic vessels, which indicates a valvular defect of lymph propulsion in collecting vessels. The deletion of *CLESR1* possibly results in valvular defects. Subsequent dysmotility or obstruction of the lymphatics in the thorax duct or intestinal lymphatic trunk leads to intestinal lymphangiectasia and PLE. It is a pity that lymphatic anomaly and dysfunction of the lymphatic flow were not demonstrated by lymphography. Primary intestinal lymphangiectasia cannot be confirmed because of the lack of endoscopic and pathologic outcomes.

The underlying molecular basis of lymphedema in 22q13.3 deletion syndrome remains to be discovered. In our patient, *CELSR1* completely included in the deletion was identified as the potential cause of the primary lymphedema and PLE. *CELSR1* is located on 22q13.3 (chr22:46756731–46933067, hg19) with a size of 176 kb and consists of 35 exons. With a relatively low residual variation intolerance score of -2.82 (0.63%), *CELSR1* is predicted to be more intolerant to functional genetic variation and more likely to be a disease-causing gene.^[12]^ It is also proposed to be intolerant of loss-of-function (LoF) variation with the probability of being LoF intolerant score of 1,^[13]^ while there is not sufficient evidence for haploinsufficiency with a haploinsufficiency score of 67.81%.^[14]^*CELSR1* haploinsufficiency is reported to be associated with lymphoedema recently.^[7,11,15]^ All reported patients from 7 pedigrees had LoF variants in *CELSR1* (p.(Glu290∗), p. (Asn681Metfs∗16), p.(I1708fs∗44), p. (Trp1957∗), c.5226+2T>A, c.5702–1G>C, c.6739+1G>A) and showed primary lymphedema in lower extremities without PLE. It was demonstrated that *CELSR1* influences vascular epithelial cell migration and proliferation,^[16]^ and *Celsr1* together with *Vangl2* functions in lymphatic valve development.^[17]^ All the above suggest that deletion of *CELSR1* may be causative for lymphedema in 2q13.3 deletion syndrome.

The distance between *CELSR1* and *SHANK3* is 4.18 Mb. We reanalyzed the data in Samogy-Costa paper^[2]^ and found that of 34 patients with 22q13.3 deletion syndrome, 15 (44.1%, 15/34) carry the deletion of *CELSR1*, while only 4 of them (4/15, 26.7%) reported lymphedema. The condition may result from incomplete penetrance. Erickson et al^[7]^ found that the manifestation of lymphedema is limited in females in a family with *CELSR1* variant and proposed the possibility of sex-limited penetrance. However, male patients with *CELSR1* deletion and lymphoedema were recorded in Samogy-Costa study,^[2]^ which conflicts with the sex-limited penetrance. We attribute the phenomena to incomplete penetrance.

In conclusion, primary lymphedema and PLE can occur in a patient with 22q13.3 deletion syndrome at the same time. The 2 phenotypes could share the same genetic etiology of congenital lymphatic abnormalities. *CELSR1* deletion may play a role in lymphatic dysplasia. The case also provides additional proof of the pathogenic effect of *CELSR1* on hereditary lymphedema.

## Acknowledgments

We acknowledge the patient and her parents for presenting their personal information.

## Author contributions

**Conceptualization:** Song Xia, Wenbin Shen.

**Funding acquisition:** Jingmin Wang.

**Investigation:** Zhong Liu.

**Methodology:** Jingmin Wang, Huifang Yan.

**Resources:** Song Xia, Kun Chang, Yuguang Sun.

**Supervision:** Wenbin Shen.

**Writing – original draft:** Zhong Liu, Huifang Yan.

**Writing – review & editing:** Song Xia, Zhong Liu, Wenbin Shen.

## References

[1] Phelan K Boccuto L RR. Phelan-McDermid Syndrome. GeneReviews® [Internet]. Seattle: University of Washington; 2005. 20301377

[2] Samogy-Costa CI Varella-Branco E Monfardini F. A Brazilian cohort of individuals with Phelan-McDermid syndrome: genotype-phenotype correlation and identification of an atypical case. J Neurodev Disord 2019;11:13 doi:10.1186/s11689-019-9273-1. 31319798 10.1186/s11689-019-9273-1 PMC6637483

[3] Kolevzon A Angarita B Bush L. Phelan-McDermid syndrome: a review of the literature and practice parameters for medical assessment and monitoring. J Neurodev Disord 2014;6:39 doi:10.1186/1866-1955-6-39. 25784960 10.1186/1866-1955-6-39 PMC4362650

[4] Sarasua SM Boccuto L Sharp JL. Clinical and genomic evaluation of 201 patients with Phelan-McDermid syndrome. Hum Genet 2014;133:847–59. doi:10.1007/s00439-014-1423-7. 24481935 10.1007/s00439-014-1423-7

[5] Connell FC Gordon K Brice G. The classification and diagnostic algorithm for primary lymphatic dysplasia: an update from 2010 to include molecular findings. Clin Genet 2013;84:303–14. 23621851 10.1111/cge.12173

[6] Umar SB DiBaise JK. Protein-losing enteropathy: case illustrations and clinical review. Am J Gastroenterol 2010;105:43–9. quiz 50. doi:10.1038/ajg.2009.561. 19789526 10.1038/ajg.2009.561

[7] Erickson RP Lai LW Mustacich DJ Bernas MJ Kuo PH Witte MH. Sex-limited penetrance of lymphedema to females with CELSR1 haploinsufficiency: a second family. Clin Genet 2019;96:478–82. doi:10.1111/cge.13622. 31403174 10.1111/cge.13622

[8] Riggs ER Andersen EF Cherry AM. Technical standards for the interpretation and reporting of constitutional copy-number variants: a joint consensus recommendation of the American College of Medical Genetics and Genomics (ACMG) and the Clinical Genome Resource (ClinGen). Genet Med 2019;doi:10.1038/s41436-019-0686-8. 10.1038/s41436-019-0686-8 PMC7313390 31690835

[9] McGaughran J Hadwen T Clark R. Progressive edema leading to pleural effusions in a female with a ring chromosome 22 leading to a 22q13 deletion. Clin Dysmorphol 2010;19:28–9. doi:10.1097/MCD.0b013e3283301f58. 19940762 10.1097/MCD.0b013e3283301f58

[10] Braamskamp MJ Dolman KM Tabbers MM. Clinical practice. Protein-losing enteropathy in children. Eur J Pediatr 2010;169:1179–85. doi:10.1007/s00431-010-1235-2. 20571826 10.1007/s00431-010-1235-2 PMC2926439

[11] Gonzalez-Garay ML Aldrich MB Rasmussen JC. A novel mutation in CELSR1 is associated with hereditary lymphedema. Vasc Cell 2016;8:01 doi:10.1186/s13221-016-0035-5. 10.1186/s13221-016-0035-5 PMC4743364 26855770

[12] Petrovski S Gussow AB Wang Q. The intolerance of regulatory sequence to genetic variation predicts gene dosage sensitivity. PLoS Genet 2015;11:e1005492 doi:10.1371/journal.pgen.1005492. 26332131 10.1371/journal.pgen.1005492 PMC4557908

[13] Lek M Karczewski KJ Minikel EV. Analysis of protein-coding genetic variation in 60,706 humans. Nature 2016;536:285–91. doi:10.1038/nature19057. 27535533 10.1038/nature19057 PMC5018207

[14] Huang N Lee I Marcotte EM Hurles ME. Characterising and predicting haploinsufficiency in the human genome. PLoS Genet 2010;6:e1001154 doi:10.1371/journal.pgen.1001154. 20976243 10.1371/journal.pgen.1001154 PMC2954820

[15] Maltese PE Michelini S Ricci M. Increasing evidence of hereditary lymphedema caused by CELSR1 loss-of-function variants. Am J Med Genet A 2019;179:1718–24. doi:10.1002/ajmg.a.61269. 31215153 10.1002/ajmg.a.61269

[16] Zhan YH Luo QC Zhang XR. CELSR1 is a positive regulator of endothelial cell migration and angiogenesis. Biochemistry (Mosc) 2016;81:591–9. doi:10.1134/S0006297916060055. 27301287 10.1134/S0006297916060055

[17] Tatin F Taddei A Weston A. Planar cell polarity protein Celsr1 regulates endothelial adherens junctions and directed cell rearrangements during valve morphogenesis. Dev Cell 2013;26:31–44. doi:10.1016/j.devcel.2013.05.015. 23792146 10.1016/j.devcel.2013.05.015 PMC3714594

